# Biconometric Connections in Dental Implants: A Pilot Mechanical Study

**DOI:** 10.3390/ma18071415

**Published:** 2025-03-23

**Authors:** Nicole Riberti, Michele Furlani, Emanuele Tognoli, Adriano Piattelli, Luca Comuzzi, Alessandra Giuliani, Andrea Gatto

**Affiliations:** 1 Neuroscience, Imaging and Clinical Sciences Department, University of Chieti-Pescara, 66100 Chieti, Italy; nicole.riberti@unich.it; 2 Biomedical Science and Public Health Department, Polytechnic University of Marche, 60131 Ancona, Italy; m.furlani@staff.univpm.it; 3 Department of Engineering “Enzo Ferrari”, University of Modena and Reggio Emilia, 41125 Modena, Italy; emanuele.tognoli@unimore.it (E.T.); agatto@unimore.it (A.G.); 4 School of Dentistry, Saint Camillus International University for Health Sciences, 00131 Rome, Italy; adriano.piattelli@unicamillus.org; 5 Independent Researcher, 31020 San Vendemiano, TV, Italy; luca.comuzzi@gmail.com; 6 Odontostomatologic and Specialized Clinical Sciences Department, Polytechnic University of Marche, 60131 Ancona, Italy

**Keywords:** dental implants, conometric connection, mechanical testing, micro-computed tomography, micro-gaps

## Abstract

Background: In dental implants, micro-gaps at the fixation–abutment interface can cause peri-implantitis and/or loosening or loss of the fixation screw; therefore, three-dimensional imaging is widely used to examine different types of connections. In the present study, we focus on the analysis on biconometric connections to detect and (possibly) measure the presence of micro-gaps in the as-positioned state and after repeated loading and unloading. Methods: Seven biconometric dental implants were characterized using micro-computed tomography (micro-CT). In two specimens (group 1), the cap was inserted, and only the apical portion was imaged, to evaluate the cap–abutment connection; in the remaining five specimens (group 2), the fixture–abutment connection was analyzed. Two implants in group 2 were also subjected to load tests to verify whether stresses could induce the formation of micro-gaps as a consequence of preload loss. Results: Micro-CT analysis showed the absence of micro-gaps greater than 10 µm in both cap–abutment and abutment–fixture connections. This was verified, in the fixture–abutment connection, even after mechanical loading and unloading. The results were reproducible in all the investigated samples in the different experimental conditions. Conclusions: In the human force range during chewing, the conical connection showed a high level of resistance to micro-gap formation at the implant–abutment interface. The absence of micro-gaps, as demonstrated here, provides encouraging preliminary data regarding the stability of the biconometric connections, which will be further verified in follow-up studies on a larger sample size.

## 1. Introduction

A mismatch between fixture and abutment in dental implants may result in two main implications: biological complications, such as peri-implantitis [1,2], and mechanical complications, such as loosening or loss of the fixation screw and, consequently, fracture of the prosthetic superstructure [3]. Therefore, a good fixture–abutment fit, together with the choice of proper materials/coatings for implant and crown manufacturing [4,5], can determine the success of the implant and good osseointegration [6].

In this context, the availability of a peri-implant mucosa of adequate size and organization certainly plays a primary role, as it allows for a correct continuum between epithelial and connective tissue, avoiding bone resorption [7,8]. Furthermore, the implant–abutment interface also controls the degree and location of peri-implant inflammatory cell accumulation, which can contribute to the extent of implant-associated alveolar bone loss [9]. Indeed, if the soft tissue size is not adequate, bone resorption occurs. Relatedly, changes in bone ridge level appear to be dependent on the location of the micro-gap [10,11].

To date, the two most-used techniques for prosthetics on endo-osseous implants are screwed and cemented ones. Screwing the prostheses onto the implants has the advantage of an easier application of the prosthesis and equally easier removal, but it has issues related to the access holes for the screwdriver, which must always be closed for esthetic reasons. This step requires the use of thin porcelain, with a risk of chipping, and it could represent an access route for bacteria that can contaminate the inner portion of the implant [12]. Conversely, the cementation of prostheses has the primary advantage of a lack of screw holes, enhancing esthetics; however, the principal disadvantage is the challenge of removing the restoration once cemented, compounded by the cement frequently accumulating beneath the gum, which is difficult to remove and serves as a prime reservoir for plaque and inflammation, often resulting in “pericementitis”. As previously noted, in cemented prostheses, the abutment is also affixed beneath the gingiva, leading to the aforementioned issues [13].

Currently, a conometric connection represents an alternative with a different rationale, as it does not involve the use of either screwing or cementation. The conometric prosthesis utilizes a tapered abutment affixed to the fixture via a Cone–Morse connection, positioned in a platform-switching manner beneath the gingival and bone profile. This is implemented during the implant surgery using the “One Abutment One Time” technique, which regulates all variables concerning the infiltration of organic fluids and tissue debris at the interface. The abutment, uniform across all implant diameters and lengths, is distinguished solely by its height/angle adjustment, to accommodate tissue thickness for superficialization and to correct disparallelisms between implants. The benefits include the absence of holes, the elimination of cement, and straightforward restoration removal. Indeed, an optimal healthy tissue appearance has been clinically observed, and proved to be sustained throughout time [14,15]. In this context, a bi-abutment connection—called “biconometry”—represents a novelty; it is made of three components: a fixture and abutment, coupled via a Cone–Morse connection, and a metal or poly-ether-ether ketone (PEEK) cap, coupled by friction to the abutment via a 4° conometric connection.

According to Schmitt et al. (2014) [16], the conical connection provides an ideal fit between the implant and the abutment, resulting in isolation of the internal part of the implant from the oral cavity. In this way, microleakage and the risk of rapid loosening are reduced. Furthermore, the load of the conical connection is transferred along the two constructions, ensuring an even distribution of forces and also providing greater stability from a mechanical point of view [16,17]. Moreover, in a literature review on different implant connections, Vinhas et al. (2020) showed that Cone–Morse connections are less prone to mechanical complications, such as fractures and screw loss [18].

Despite the success demonstrated by conical connections in clinical practice, the incidental presence of micro-gaps continues to be repeatedly investigated within different frameworks and at different resolutions. Among these, worthy of mention is a study that used synchrotron radiation to analyze, during compression tests up to 100 N, the mechanical behavior in the coupling area of conical implants, also attempting to detect micro-movements [19]. Another study recorded, by means of a high-resolution and high-speed X-ray camera, a chewing simulator with dynamic and static loading up to 200 N on twenty implants, some with conical implant–abutment connections and others with flat connections; the conical connections did not show micro-gaps, while some micro-movements were found for the flat connections, both in static and dynamic loading conditions [20]. Other studies showed that crestal bone alterations around titanium implants are considerably influenced by potential movements between implants and abutments, but not by micro-gap size [21]. Some other groups investigated preload loss and bacterial penetration at the implant–abutment interface of conical and internal–external hexagon connection systems exposed to temperature cycling and mechanical wear, in order to determine the influence of implant geometry on bacterial penetration and load deformations as a result of testing different models [22,23,24,25].

In this context, micro-computed tomography (micro-CT) has allowed for studying the effectiveness of the seal between the implant and abutment in different settings [26,27,28,29]; indeed, it was recently demonstrated by synchrotron-based micro-CT that adequate and well-structured connective tissue surrounded the abutment in a conometric system implanted in a patient. Specifically, the implant’s effectiveness was supported by a quantitative assessment of the connective tissue achieved by artificial intelligence tools, featuring interlaced longitudinal and transverse collagen bundles [30].

The present study aims to detect and measure, by micro-CT, the possible presence of micro-gaps in biconometric connections, before and after application of a series of two successive load tests, the first up to 150 N and the second up to 300 N. Indeed, beyond the above-mentioned successful clinical results shared in the reference literature, this study is necessary to start a systematic investigation into the mechanical performance of biconometric connections, which have so far been marginally treated in the literature.

## 2. Materials and Methods

### 2.1. Experiment Workflow

Tests to detect the possible presence of micro-gaps were carried out by micro-CT analysis.

**Figure 1:**
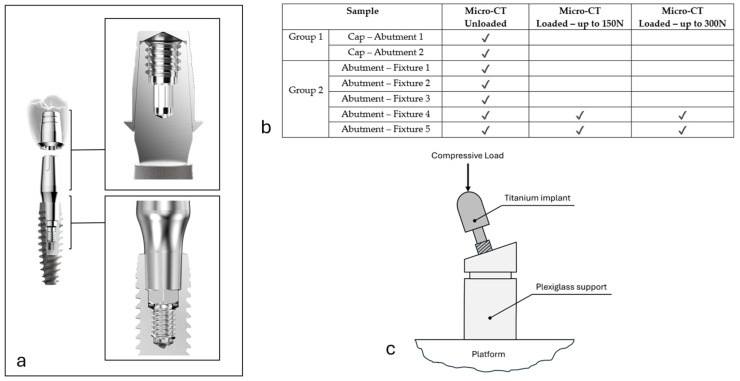
(**a**) Biconometric connection made of three components: fixture, abutment, and cap; (**b**) table summarizing groups and sample size; (**c**) scheme of titanium implant screwed in plexiglass support and subjected to external compressive load. Arrow indicates direction in which load was applied.

In the first phase of the study, a total of seven (*n = 7*) implant samples were acquired, each of them screwed in plexiglass in the basal part (Figure 1). In two (*n* = 2) dental implants (group 1), the cap was inserted; here, only the apical portion was acquired, to evaluate the junction with the underlying abutment. In the remaining five (*n = 5*) samples (group 2), the connection portion between the fixture and the abutment was analyzed.

In the second phase of the study, two (*n = 2*) implants of group 2 were also subjected to mechanical tests. In order to reproduce in-service conditions, these samples were screwed in plexiglass at an angle of inclination of 60° relative to the horizontal plane (Figure 1). A compression test was performed twice on each sample: the first test was conducted up to a load of 150 N, while the second was conducted up to 300 N. After each load test, a micro-CT scan was performed to detect and eventually measure the presence of micro-gaps at the fixture/abutment connection post-compression up to 150 N and post-compression up to 300 N, as a result of the relative displacement generated by the load cycle.

A table summarizing the groups and sample size is reported in Figure 1b.

### 2.2. Implant Manufacturing

The investigated biconometric dental implants (AoN Implants Srl, Grisignano di Zocco (VI)—Italy) were endo-osseous screws designed for dental surgery; they can be implanted in previously prepared sites of edentulous areas, according to specific operative sequences.

A biconometric connection (Figure 1a) is made up of three components: (1) the implant (or fixture); (2) the abutment, which is coupled to the implant via a Cone–Morse connection; (3) the cap, coupled by friction to the abutment via a 4° conometric connection.

The material used for the implant production was cold-drawn Gr. 4 Titanium; it was selected for its strength and because it is not contaminated by other elements. The machining process was carried out through numerically controlled machinery, nominally maintaining control down to 1 µm details. During production, the produced components were checked by the systems VICI Vision (Vici & C S.p.A., Santarcangelo di Romagna (RN), Italy) and Hexagon OPTIV M (Hexagon Metrology Spa, Grugliasco (TO), Italy). The body of the implant was structured with a two-main loop so that, with each revolution, the screw advanced twice as much as a classic pitch implant, without creating stress in the recipient bone.

Afterwards, in the post-production checks, thorough cleaning was performed to completely degrease the parts with an ultrasonic metal washer (ILSA-MC Srl, San Pietro in Casale (BO), Italy); moreover, each component was checked for quality by visual inspection and centesimal calipers, micrometers, pin calipers, and internal and external thread calipers. Subsequently, a final cleaning process was performed, and the product was bagged in medical paper.

The abutment, made of Gr. 5 Titanium, was washed and checked as described for the implant; furthermore, the prosthetic cap, also made of Gr. 5 Titanium, was checked with the previously described instruments.

The abutment–implant coupling was performed by fixing the screw at 30 Ncm, as specified in the manufacturer’s instructions, while the cap–abutment coupling was performed using a calibrated instrument by applying a force of 30 Ncm axially to the head of the cap, as previously reported [31].

### 2.3. X-Ray Micro-CT Analysis

The Metro-Tom 1500 ZEISS (Carl Zeiss Industrielle Messtechnik GmbH, Oberkochen, Germany) was used for three-dimensional (3D) micro-CT analysis, setting the X-ray source with a voltage at 200 kV and a current at 40 µA. The pixel size was set to 7.93 µm. An aluminum filter, 1.5 mm thick, was introduced to select photons in the high-energy range; finally, two hours of scanning time were spent for each specimen, with a total of 2050 projections acquired per sample. The image reconstruction was automatically performed by the Metrotom os (vers. 3.0) software.

### 2.4. Image Segmentation, Thickness Map, and Image Registration

All images were elaborated with the Dragonfly software (Dragonfly, vers. 2022.2, Comet Technologies Canada Inc., Montreal, QC, Canada; software available at https://dragonfly.comet.tech/, accessed on 21 March 2025) [32]. The software was used in the image processing phase; it allowed for a search for micro-gaps at the cap–abutment and fixture–abutment connections, segmenting the different components of the samples and creating thickness maps capable of detecting any internal micro-gap caused by relative misalignment after the compression tests. The software has the capability to calculate and create color-coded meshes that display the reference values of the local thickness between boundary points, which is useful for visualization and analysis. The thickness was determined by measuring the maximum diameter of an imaginary sphere that could be inserted inside the boundary walls.

Registration between the 3D reconstructions performed after the 150 N load and the one after the 300 N load was performed using the Dragonfly Image Registration tool. This software module can automatically register datasets by applying the scaling, rotations, and translations required to match features between two datasets. Two matching processes are made available by the software for registering datasets: the Mutual Information and the SSD (sum of squared differences). These methods are based on different concepts to quantify the degree of similarity between images and to apply the required linear transformations. The results may differ significantly: the performance of each algorithm is also dependent on the input data and the mode (Basic or Advanced) selected for registration. After several trials, we chose the SSD method in the Advanced mode.

### 2.5. Mechanical Compression Tests

The compression tests were performed on an INSTRON 5567 machine (Instron, Norwood, MA, USA) equipped with a load cell with a maximum load capacity of 1 kN. The speed of the crosshead was kept constant at a value of 5 mm/min during the test. Both samples were tested twice, with a first compression test up to a maximum load of 150 N, and a second compression test up to a maximum load of 300 N. At the end of each test, the sample was unloaded immediately after reaching the maximum load. The load was applied directly to the upper part of the implant, in a direction perpendicular to the base of the sample (Figure 1c). The curves of the tests were determined as the compressive displacement (X-axis) and compressive load (Y-axis) for both tested samples.

## 3. Results

### 3.1. Micro-CT Analysis

**Figure 2:**
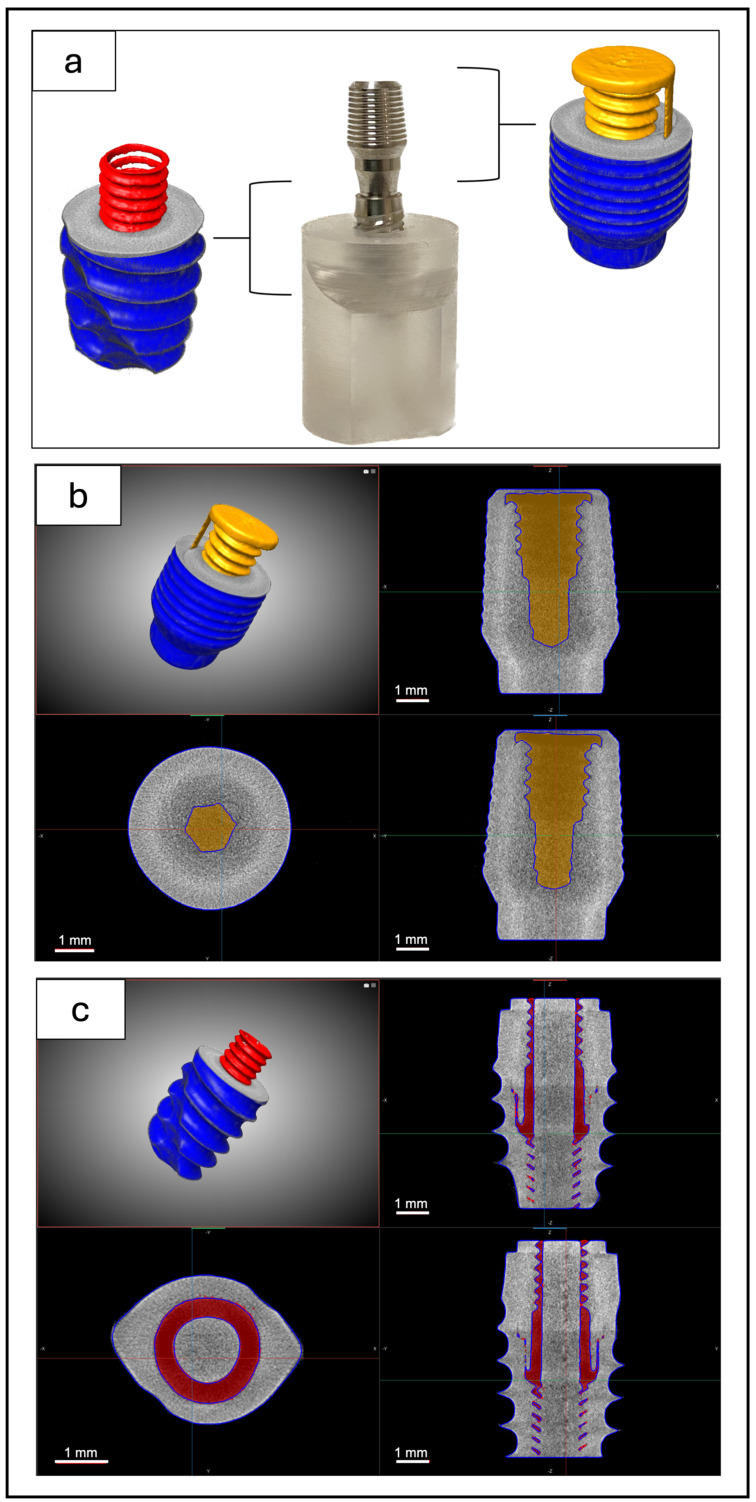
(**a**) Micro-CT 3D reconstructions: different portions of representative samples imaged in static conditions after preload at 30 Ncm; (**b**) apical portion: cap–abutment connection; (**c**) implant–abutment connection. Portions rendered in yellow or red represent structural voids inside samples, respectively, in apical portion or in implant–abutment junction.

Figure 2 shows the different portions acquired by micro-CT before the mechanical compression tests. In the top panels (Figure 2b), the connection of the cap to the abutment is imaged, while the implant–abutment connection is observed in the bottom panel (Figure 2c). The portions rendered in yellow or red represent the structural voids inside the sample, respectively, in the apical portion or in the implant–abutment junction.

The sharp difference in gray tones between the background (i.e., voids) and the titanium material allowed for segmentation of the brightness histograms using the thresholding method. From the highlighted voids and according to the thickness analysis performed, there was no evidence of micro-gaps either between the implant and abutment or between the abutment and cap. Therefore, at the present experimental resolution, the presence of micro-gaps of size >10 µm, after the preload at 30 Ncm, can be excluded in both cap–abutment and abutment–fixture connections.

All micro-CT analyses performed on the samples before compressive loading excluded the presence of micro-gaps both in the two samples of group 1, referring to the study of the cap–abutment connection, and in the five samples of group 2, referring to the study of the fixture–abutment connection.

As a second experimental step, the 3D scans were repeated on two samples of group 2, both after compression up to a 150 N load, and after compression up to a 300 N load. The vertical compression on the implant did not produce evident signs of permanent micro-gaps due to a loss of preload or misalignment. In fact, no micro-gaps were recorded at the implant–abutment connection in either of the two samples, neither after reaching the load of 150 N, nor after loading up to 300 N. To further confirm this result, a registration was performed between the 3D reconstructions after the 150 N load and those after the 300 N load using the Image Registration Dragonfly’s feature-based registration workflows.

**Figure 3:**
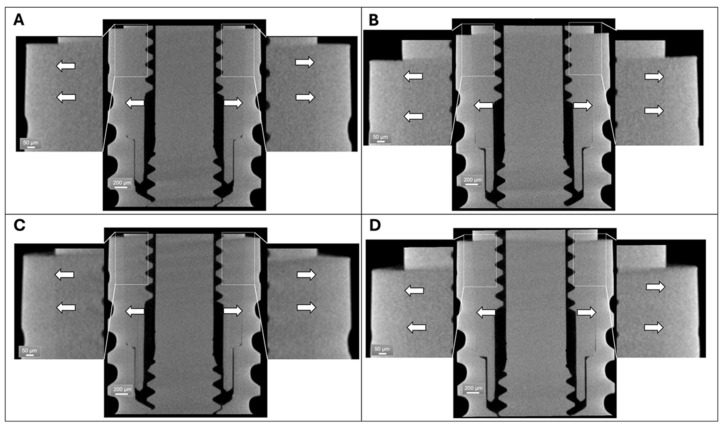
Micro-CT reconstruction in sagittal section of (**A**) Sample 1, compressed up to 150 N; (**B**) Sample 2, compressed up to 150 N; (**C**) Sample 1, compressed up to 300 N; and (**D**) Sample 2, compressed up to 300 N. White arrows: abutment – fixture interface.

The registration outputs gave no evident sign of decoupling at the fixture–abutment connection, as shown by the white arrows in Figure 3. In particular, no micro-gaps were found at the fixture–abutment connection for both the samples after the two compression processes, as displayed in the magnifications of Figure 3.

### 3.2. Mechanical Tests

**Figure 4:**
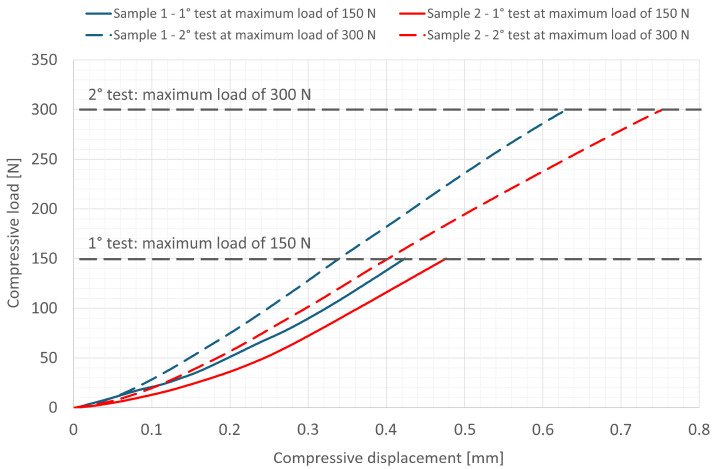
Load–displacement compression curves. Curves are shown for each sample tested, up to maximum load of 150 N (full lines) and 300 N (dotted lines), respectively.

The load–displacement compression curves were registered for each test performed (Figure 4). The curves reveal, in both samples, that the tests performed up to a maximum load of 300 N have a greater slope than the previous tests carried out—on the same samples—up to a maximum load of 150 N. In addition, for both applied loads, a deviation can be observed between the curves of the two samples tested, greater than the possible uncertainty of the machine. This phenomenon can be explained by the fact that the preparation of the samples was a manual process, which could have led to slight differences between each sample. Finally, in all the tests performed, the components did not reach their maximum load bearing ability and remained structurally intact, with no visible cracks or damage after the load had been removed.

## 4. Discussion

In this study, the focus is on the two different connections present within a biconometric dental implant: the fixture–abutment connection and the prosthetic cap–abutment connection.

Several clinical studies on this prosthetic technique have been performed [14,15,33,34,35,36,37,38]; all of these authors reported extremely satisfactory clinical data with the use of this technique, with follow-up ranging from 1 up to 5 years. Two microbiological studies have been conducted to evaluate bacterial infiltration at the level of the caps [39,40], in which no bacterial infiltration was found.

Other studies have reported that the functional load transmission to the fixture is influenced by the abutment connection area’s design [41,42,43,44]. Implants with different internal connections (for example, hexagons and octagons) were subjected to cyclic load tests, and it was shown that connections approximating circular shapes are less prone to failure [44]. Furthermore, the materials and production processes of the implant can influence the presence of micro-gaps and marginal bone loss [45,46]. In particular, implants with different mismatching distances and different prosthetic abutment heights may have different effects with regard to bacterial infiltration and structural fracture of the implant.

For this reason, in the present study, innovative biconometric dental implants, characterized by the selection of Gr. 4 Titanium, with processes controlled at each step at the micrometric level of detail [47], were analyzed using the micro-CT imaging technique to verify the stability of the connections after compressive loading.

Although mechanical tests showed, in both specimens, a steeper slope of the load/displacement curve in the second test, i.e., up to a maximum load of 300 N, compared to tests performed up to a maximum load of 150 N, both specimens showed no decoupling (micro-gaps), i.e., loosening of connection, after the removal of the weight. Moreover, as the components did not reach the compressive load limit, they remained structurally intact, with no fractures, obvious cracks, or damage.

Furthermore, micro-CT allowed for imaging of the samples in three dimensions, achieving evaluation of the internal connections and interfaces between the different components in both of the groups. The acquisition pixel size was set to 7.93 µm, which means that micro-gaps with dimensions <10 µm were not detectable. However, this level of resolution is still reliable for evaluating the quality of the connections, since the micro-gaps that determine an actual instability of the coupling during chewing are in this dimensional range, ultimately producing two effects: the first is biological, creating and amplifying the inflammation of the soft tissues and favoring bacterial invasion; the second is structural, causing possible microfractures of the implant crown, of the implant itself, or of the peri-implant bone.

Acquisitions before use, in the as-implanted condition, were performed to evaluate the quality of the connections just after the preload of the connections at 30 Ncm, while acquisitions following the mechanical tests allowed for documentation of the quality of the connections after a use phase. During the virtual dissection of the 3D images of the samples, no micro-gaps were observed in the apical regions, considered the most critical due to their greater exposure to the apex of the implant. Consequently, the presence of micro-gaps with dimensions >10 µm was excluded. Such good performances are most likely related to the connection concept, which facilitates greater contact between the implant and the abutment, thus mitigating abutment instability (flexion instability), tissue inflammation, bacterial infiltration, and potential implant failure.

The results of the present study support and justify the successful clinical performance of these biconometric implants, already shown in the literature [30,33,35]. Indeed, many surgical and prosthetic benefits for the surgeon, prosthetist, and patient have been documented. For instance, the surgical benefit of sub-crestal implant placement at 1–2 mm was related to the preservation of the implant within the bone envelope, creating additional vertical space for the prosthetist, while also providing a mechanically stable connection that inhibits inflammatory infiltration and bacterial colonization [39,40]. The prosthetist utilizes tapered abutments and hoods to leverage the coupling between components, eliminating the need for cement and screws, and hence enhancing precision while minimizing bulk and ensuring adequate vertical space; this is nearly always achievable. The patient experiences definite outcomes, achieved rapidly; during dental hygiene sessions, plaque removal is easily accomplished at will, and chairside seating is expedited. Additional benefits encompass the utilization of minimal prosthetic components, hence substantially reducing implementation costs and streamlining all operations.

Our observations support the outcome of a previous study, where different connections were compared; indeed, conical connections resulted in reduced microleakage at the implant–abutment interface compared to parallel connections [24]. Moreover, conical connections appeared to have better performance, exhibiting less peri-implant bone loss; thus, crestal bone levels are more effectively preserved [48].

However, despite the good results achieved, this pilot study presents some limitations that will be partially overcome in the follow-up studies.

The resolution of the CT measurements limits the study to gaps larger than 10 µm, but this threshold should be evaluated against the dimensional accuracy expected to characterize the specific application. In particular, the study involves experimental conditions that replicate a clinical setting where the dimensional deviations are affected by the following: (i) the typical manufacturing tolerance; (ii) the typical positioning tolerance during the surgical phase; (iii) the thermal deformation due to the temperature in the mouth (which is different from the test temperature); and (iv) the different stiffness and consequent differential deformation of the implant fixture and the biological tissues (bone, connective tissue, etc.). All of these factors add up in a chain of tolerances to an expected deviation of more than 10 µm.

Moreover, in our study, bone was modeled with plexiglass. The different stiffness measured during the two loading phases and observed in Figure 4 could very likely be attributable to the plexiglass undergoing changes as a result of loading. This effect will be the subject of follow-up studies to evaluate only the mechanical response of the plexiglass alone, to decouple it from the response of the entire construct.

Other limitations of this study include the limited sample size and loading steps. Indeed, the force generated during routine chewing of foods such as carrots or meat is about 70–150 N. The maximum chewing force in some people can reach up to 500–700 N [49]. Therefore, we selected two values within the range and spaced by 150 N; however, we plan to add two steps, namely 450 N and 600 N, in follow-up studies. Furthermore, regarding the resolution capacity of the tomographic instrumentation, it will be useful to use tomographs capable of reaching higher resolutions, in order to identify and measure even smaller gaps at the fixture/abutment interface.

## 5. Conclusions

Within the limits of the small sample size, our investigation revealed that, within the range of human force during chewing, the conical connection showed high resistance against the creation of micro-gaps at the implant–abutment and abutment–prosthesis interfaces. The absence of micro-gaps in biconometric implants, at micrometric resolution, as demonstrated here, provides encouraging preliminary data regarding the stability of the biconometric connections, which will be further verified in follow-up studies on a larger sample size.

## Data Availability

The original contributions presented in this study are included in the article. Further inquiries can be directed to the corresponding author.

## Abbreviations

The following abbreviations are used in this manuscript:

Micro-CT	Micro-computed tomography
